# Job satisfaction of the nursing team in intensive care units

**DOI:** 10.1590/1518-8345.3168.3182

**Published:** 2019-10-07

**Authors:** Kelly Yukari Teruya, Ana Cláudia de Souza Costa, Edinêis de Brito Guirardello

**Affiliations:** 1 Universidade Estadual de Campinas, Faculdade de Enfermagem, Campinas, SP, Brazil.; 2 Scholarship holder at Programa Institucional de Bolsas de Iniciação Científica da Unicamp (PIBIC/PIBITI), Brazil.; 3 Pontifícia Universidade Católica de Minas Gerais, Poços de Caldas, MG, Brazil.

**Keywords:** Job Satisfaction, Intensive Care Units, Nursing, Health Facility Environment, Critical Care, Nursing Service, Hospital, Satisfação no Emprego, Unidades de Terapia Intensiva, Enfermagem, Ambiente de Instituições de Saúde, Cuidados Críticos, Serviço Hospitalar de Enfermagem, Satisfacción en el Trabajo, Unidades de Cuidados Intensivos, Enfermería, Ambiente de Instituciones de Salud, Cuidados Críticos, Servicio de Enfermería en Hospital

## Abstract

**Objective:**

to evaluate job satisfaction and its relationship with the personal and professional characteristics of the nursing team.

**Method:**

a descriptive and cross-sectional study with 163 nursing workers from the intensive care units of a teaching hospital. For data collection, the Brazilian version of the Job Satisfaction Survey and a personal and professional characterization form were used. Data were analyzed using descriptive statistics, comparisons and correlations.

**Results:**

the professionals demonstrated ambivalence for job satisfaction in a global way and concerning the communication domain. They were satisfied with the supervision, co-workers, and nature of work, while dissatisfied with other domains. There was a correlation between the intention to stay in the job and the majority of the Job Satisfaction Survey domains, except for co-workers and operating procedures, and a correlation between time working at the unit and at the institution with the domains pay, contingent rewards, and supervision.

**Conclusion:**

there was an ambivalence regarding job satisfaction and the variables intention of stay in the job and time working at the unit and at the institution were correlated with job satisfaction concerning the domains pay, contingent rewards, and supervision.

## Abstract (pt)

**Objetivo:**

avaliar a satisfação no trabalho e sua relação com as características pessoais e profissionais da equipe de enfermagem.

**Método:**

estudo descritivo e transversal com 163 trabalhadores da enfermagem de unidades de terapia intensiva de um hospital de ensino. Para a coleta de dados, foi utilizada a versão brasileira do Job Satisfaction Survey e uma ficha de caracterização pessoal e profissional. Analisaram-se os dados por meio de estatística descritiva, comparações e correlações.

**Resultados:**

os profissionais demonstraram ambivalência para satisfação no trabalho de forma global e para o domínio comunicação. Estavam satisfeitos no que tange a supervisão, colaboradores e natureza do trabalho e insatisfeitos quanto aos demais domínios. Houve correlação entre intenção de permanecer no trabalho e a maioria dos domínios do Job Satisfaction Survey, com exceção de colaboradores e condições operacionais, e entre o tempo de trabalho na unidade, e na instituição, com os domínios remuneração, recompensas e supervisão.

**Conclusão:**

houve ambivalência em relação à satisfação no trabalho e quanto às variáveis intenção de permanecer no trabalho e tempo de trabalho na unidade e na instituição apresentaram correlação com satisfação profissional para os domínios remuneração, recompensa e supervisão.

## Abstract (es)

**Objetivo:**

avaliar la satisfacción en el trabajo y su relación con las características personales y profesionales del equipo de enfermería.

**Método:**

estudio descriptivo y transversal con 163 trabajadores de enfermería de unidades de terapia intensiva en un hospital escuela. Para la recolección de datos, se utilizó la versión brasileña del Job Satisfaction Survey y una ficha de caracterización personal y profesional. Se analizaron los datos por medio de estadística descriptiva, comparaciones y correlaciones.

**Resultados:**

los profesionales mostraron ambivalencia para la satisfacción en el trabajo de forma global y para el dominio de la comunicación. Estaban satisfechos por lo que se refiere a la supervisión, colaboradores y naturaleza del trabajo, e insatisfechos en cuanto a los demás dominios. Se observó correlación entre la intención de permanecer en el trabajo y la mayoría de los dominios del Job Satisfaction Survey, excepto de los dominios colaboradores y condiciones operativas, y la correlación entre el tiempo de trabajo en la unidad y en la institución con los ámbitos de la remuneración, las recompensas y la supervisión.

**Conclusión:**

Se verificó ambivalencia en relación a la satisfacción y las variables intención de permanecer en el trabajo, y tiempo de trabajo en la unidad y en la institución se correlacionaron con satisfacción en el trabajo para los dominios remuneración, recompensa y supervisión.

## Introduction

Job satisfaction is a complex phenomenon with multiple causal factors related to the work environment, supervision, and management. It is defined as the positive response of professionals to working conditions that meet their needs, as a result of their assessment of the value or fairness of their professional experience^(^ 1 ^)^.

Benefits and salary compensations are the factors that most influence the satisfaction of nursing professionals^(^ 2 ^)^, followed by workload, recognition, institutional incentive^(^ 3 ^-^ 4 ^)^, autonomy, and respect of the colleagues^(^ 5 ^)^. On the other hand, job satisfaction influences the intention to stay in the job^(^ 6 ^)^ and in the institution^(^ 7 ^)^, as well as reflects on the quality and safety of nursing care^(^ 8 ^)^.

In a critical care environment, the continuous presence of suffering and death, highly complex care, and the use of advanced technologies, among other elements, may lead to professional dissatisfaction and hinder the quality of life at work of nursing professionals^(^ 9 ^)^. The practice of leadership and organizational commitment were predictors for job satisfaction of nurses in critical care units^(^ 10 ^)^, as well as workload^(^ 11 ^)^, relationship with the work team, autonomy, compensation, and recognition^(^ 12 ^-^ 13 ^)^.

It is an environment that needs constant updating of professionals concerning technological innovations and complexity of patient care, as well as management support to deal with conflicts and develop interdisciplinary actions in the health context, thus promoting safe care. In addition, nursing is the largest category of health professionals, responsible for continuous care to patients, which reinforces the need to value an environment in which professionals may be satisfied with their work, exercising their activities with quality and ensuring the safety of patients. Therefore, this study aimed to evaluate job satisfaction and its relationship with the personal and professional characteristics of the nursing team.

## Method

This is a correlational and cross-sectional study, carried out in three intensive care units (ICU) of a teaching institution that belongs to the Brazilian public health network, located in the countryside of the state of São Paulo. It is a large and of high complexity hospital, whose ICUs are located in three different physical structures. The first is called General ICU, containing postoperative, neurology, and coronary units. The second is named Trauma and Clinical ICU, which serves the specialties of Trauma and Internal Medicine. Finally, the third is Transplant ICU.

The nursing team consists of nurses, nursing technicians, supervisors, and the director. However, for this study, we only considered nurses and nursing technicians who provide direct care to patients and with experience time at the unit equal to or greater than three months. This is a convenience sample. Exclusion criteria were: working in the management area or being on vacation, leave of absence, or other types of absence during the data collection period.

The study was approved by the Ethics Committee (No. 2.237.564) and followed resolution 466/12 of the Brazilian Health National Council for research involving human beings. All participants signed the informed consent form.

Data collection was performed in the months of November and December 2017. For those who agreed to participate in the study, an envelope containing the instruments and the consent form was delivered. Thus, the participants filled a personal and professional characterization form and answered the Brazilian version of the Job Satisfaction Survey (JSS)^(^ 14 ^)^. The personal and professional characterization form contained the variables age, sex, marital status, professional category, professional qualification, unit, work shift, experience time in nursing, time working at the unit and at the institution, existence of another employment relationship, and intention of remaining in the job the following year, on a visual scale of zero to ten, the higher the score, the greater the intention to stay in the job.

The Brazilian version of the JSS has the purpose of evaluating the individual’s satisfaction with his/her job. It consists of 32 items and nine domains: pay (four items), benefits (three items), promotion (three items), co-workers (four items), contingent rewards (four items), nature of work (three items), supervision (four items), operating procedures (three items), and communication (four items). The measurement scale is a six-point Likert, varying from “disagree strongly” (one point) to “agree strongly” (six points) - the higher the score, the greater the job satisfaction. The values of the Cronbach’s alpha coefficient of the JSS domains ranged from 0.50 to 0.74^(^ 14 ^)^.

It should be mentioned that, in the data analysis, for the domains benefits, promotion, nature of work, and operating procedures, we considered as job satisfaction mean values between 12 and 18 points; dissatisfaction for means between three and nine points; and for values between 10 and 11, it was considered that they were neither satisfied or dissatisfied with the job, which is called ambivalence. For the domains pay, contingent rewards, supervision, and communication, mean values between 16 and 24 points indicated satisfaction; between four and 12 points, dissatisfaction; and means between 13 and 15 points represented ambivalence^(^ 14 ^)^.

For this study, the internal consistency of the JSS subscales, assessed using the Cronbach’s alpha coefficient was, for each domain: supervision (α=0.81), promotion (α=0.74), co-workers (α=0.66), communication (α=0.63), contingent rewards (α=0.62), nature of work (α=0.61), pay (α=0.59), benefits (α=0.57), and operating procedures (α=0.33). The collected data were inserted into a spreadsheet of the Microsoft Office Excel 2013 program and subjected to analysis by a statistician.

To analyze JSS subscales and the personal and professional variables, descriptive analysis was carried out. For the comparisons between a qualitative variable with two categories and a quantitative variable, we used the non-parametric Mann-Whitney test or unpaired Student’s t-test, according to the data distribution. For comparisons between a qualitative variable with more than two categories and a quantitative variable, we used the Anova model followed by Tukey’s post-test, or the non-parametric Kruskal Wallis test followed by Dunn’s post-test, according to the data distribution.

The correlations between the subscales of the JSS and the other variables in the study were verified by Spearman’s correlation coefficient, which varies from -1 to 1, according to the following classification: absence of correlation (0.00), weak correlation (0.10-0.29), moderate correlation (0.30-0.49), and strong correlation (≥0.5)^(^ 15 ^)^. For all analyses, the statistical software SAS^®^ version 9.4 and SPSS^®^ version 22 were used. For the statistical tests, a significance level of 5% was considered.

## Results

The study included 163 nursing professionals, with a mean age of 38.7 years (± 8.6), and a response rate of 93.34%. The sample characterization data are presented in Table 1.

**Table 1:** Description of the characteristics of the sample. Campinas, SP, Brazil, 2017

Variable	n	%
Sex		
Female	123	75.46
Male	40	24.54
Marital status		
Single	51	31.48
Married/married de facto	91	56.17
Separated/divorced	20	12.35
No information	1	
Professional category		
Nurse	49	30.06
Nursing technician	114	69.94
Professional Qualification		
*Stricto Sensu* Master's/PhD	5	3.07
*Lato Sensu* Upgrading/Residency	2	1.22
Specialization	35	21.47
Intensive Care Unit		
Post-operative/Neurology/Coronary	74	45.40
Trauma and Clinical	65	39.88
Transplant	24	14.72
Shift		
Morning	44	26.99
Afternoon	42	25.77
Night	77	47.24
Other employment		
Yes	36	22.09
No	127	77.91

The mean time of professional experience was 14.9 years (±7.6), the mean time working at the unit was seven years (±5.5) and the mean time working at the institution was 9.6 years (±7.1). As for the intention to stay in the current job in the next year, the mean was 8.5 (±2.7), on a scale of zero to ten points. When evaluating the personal and professional variables, we verified that the Post-operative/Neurology/Coronary and Trauma and Clinical ICUs differ regarding time working at the unit (p=0.0222).

Regarding the assessment of job satisfaction, we obtained a mean value of 107.98 (±33.9) for the total score, indicating ambivalence. Regarding the domains, the mean values ranged from 5.68 to 16.81 (Table 2), demonstrating that workers are satisfied with supervision, co-workers, and nature of work; ambivalent concerning communication; and dissatisfied with contingent rewards, pay, benefits, operating procedures, and promotion.

**Table 2:** Descriptive analysis of job satisfaction for the domains of the Job Satisfaction Survey - Brazilian version (n=163). Campinas, SP, Brazil, 2017

Job satisfaction	Mean	Standard Deviation	Minimum	First Quartile	Median	Third Quartile	Maximum
Supervision	16.81	4.78	4.00	14.00	17.00	20.00	24.00
Co-workers	16.04	3.58	4.00	14.00	16.00	18.00	24.00
Nature of work	14.41	2.91	5.00	13.00	15.00	17.00	18.00
Communication	13.55	4.24	4.00	10.00	14.00	16.00	24.00
Contingent rewards	11.38	4.33	4.00	8.00	12.00	14.00	24.00
Pay	10.67	4.26	4.00	8.00	11.00	14.00	21.00
Benefits	9.96	3.71	3.00	8.00	10.00	12.00	18.00
Operating procedures	9.48	2.84	3.00	8.00	10.00	11.00	17.00
Promotion	5.68	3.28	3.00	3.00	4.00	8.00	18.00

Next, we evaluated whether the perception of job satisfaction differs among personal and professional characteristics. There were differences between the sexes for the domain nature of work (p=0.0251); between professional categories concerning the domains contingent rewards (p=0.0228), nature of work (p=0.0158), operating procedures (p<0.0001), and communication (p=0.0442); and between ICUs for the domain communication (p=0.0242). Comparing the work shift, there was a statistically significant difference between the three periods concerning pay (p=0.0186), benefits (p=0.0187), and contingent rewards (p=0.0220), with the afternoon period obtaining higher means of job satisfaction, followed by the night and morning shifts. However, for these three subscales, the comparison was statistically significant only between the morning and afternoon shifts. The evaluation of the existence of a correlation between the JSS domains and other personal and professional variables is presented in Table 3.

**Table 3:** Spearman correlation coefficient between the subscales of the Job Satisfaction Survey - Brazilian version and personal and professional variables. Campinas, SP, Brazil, 2017

Job subscales Satisfaction Survey	Age	Experience time	Time working at the unit	Time working at the institution	Intention of remaining
Pay	-0.01	-0.06	-0.22*	-0.18*	0.31^†^
Benefits	-0.06	-0.01	-0.09	-0.08	0.19*
Promotion	0.12	0.05	-0.11	-0.11	0.24*
Co-workers	0.09	0.09	0.00	0.04	0.10
Contingent rewards	-0.00	-0.09	-0.24*	-0.22*	0.39^†^
Nature of work	0.17	0.04	0.09	0.03	0.20*
Supervision	-0.12	-0.15	0.25*	-0.24*	0.24*
Operating procedures	-0.03	-0.00	0.04	-0.00	-0.02
Communication	0.02	-0.06	-0.05	-0.03	0.16*

* p < 0.05;

† p < 0.0001

## Discussion

This study sought to evaluate job satisfaction and its relationship with personal and professional variables of the nursing team at an ICU. The sample was mainly represented by young adult women, married, and with only one employment relationship.

Most nurses have specialization, but not all related to their area of expertise. Although specialization in ICU is not a prerequisite for the practice of the profession, we verified that nurses show concern in the continuous search for training and knowledge updating.

The professionals reported a mean experience time at the unit of over seven years, and those who worked in the Post-operative/Neurology/Coronary units had a longer experience compared to the professionals of the Trauma and Clinical ICU. The experience time depicts a team with the appropriate level of knowledge and skills to act in such complex units, as is the case of intensive care units. As in another study on the subject, the mean value for the variable intention to stay in the job was high, indicating that the professionals intend to continue in the current job^(^ 16 ^)^.

JSS enables the assessment of satisfaction in a general way, considering the total number of items, as well as separately, by the domains. In the analysis, considering the total score, the professionals were not satisfied or dissatisfied with their jobs, which can be understood as a perception of ambivalence for job satisfaction. However, these findings cannot be compared with other studies in Brazil, since this is the first study that evaluated the job satisfaction of the nursing team using the Brazilian version of the JSS. However, in a study carried out with nurses in Saudi Arabia, ambivalence was also reported regarding job satisfaction^(^ 17 ^)^.

However, when assessing satisfaction by the JSS domains, satisfaction was found with supervision, co-workers, and the nature of work, which was also identified in other international studies^(^ 18 ^-^ 19 ^)^. On the other hand, the professionals showed dissatisfaction with most JSS domains, which were: contingent rewards, pay, benefits, operating procedures, and promotion. The perception of dissatisfaction expressed in the contingent rewards and operating procedures domains was also reported by nurses in Saudi Arabia^(^ 17 ^)^, while for nurses in Turkey dissatisfaction was attributed to contingent rewards, pay, and benefits^(^ 19 ^)^.

Some studies that evaluated job satisfaction using other instruments, other than JSS, also evidenced that benefits^(^ 4 ^)^ and pay^(^ 20 ^)^ negatively influenced the perception of job satisfaction. It is important to highlight that, in this study, the professionals reported ambivalence only for the communication domain, which differs from the Saudi nurses, for whom most domains were perceived as ambivalent^(^ 17 ^)^.

As for sex, men were more satisfied than women, regarding the domain nature of work, corroborating data from the national literature in which men presented higher rates of job satisfaction^(^ 21 ^)^. In international research, women presented higher levels of job satisfaction than men^(^ 22 ^)^.

Nursing professionals also differed among categories regarding job satisfaction, with nursing technicians being more satisfied than nurses in the domains: contingent rewards, nature of work, operating procedures, and communication. Similar data were found in a study that used the Index of Work Satisfaction and revealed that nursing technicians were more satisfied than nurses^(^ 23 ^)^.

The professionals of the Transplants ICU were more satisfied for the communication domain, compared to the professionals of the Trauma and Clinical ICU, which can be justified by the profile of patients and the composition of the multidisciplinary team. Communication in complex environments becomes a challenge for these professionals, mainly due to the need of establishing clear and secure communication with the multidisciplinary team, to assist those patients^(^ 24 ^)^.

Work shift also influenced the perception of satisfaction, since the professionals who worked in the afternoon were more satisfied, as demonstrated by the domains pay, benefits, and contingent rewards compared to the morning shift. It is interesting to highlight that most professionals who worked in the afternoon shift, besides being younger, had less time working at the unit and at the institution and this feeling of satisfaction may be explained by the respective domains already mentioned, which motivated them in the pursuit and permanence in the current job.

Regarding the correlation between the JSS domains with the variables age, time of professional experience, time working at the unit and at the institution, we verified: positive correlation of weak magnitude between age and nature of work; negative correlation of weak magnitude between time working at the unit and at the institution and the domains pay, contingent rewards, and supervision; positive correlation of moderate magnitude between intention to stay in the job and the domains pay and contingent rewards; and positive correlation of weak magnitude with the domains benefits, promotion, nature of work, supervision, and communication. These findings are similar to the correlation study carried out in Turkey, in which job satisfaction was directly related to age and inversely related to intention to quit the job^(^ 19 ^)^.

The intention to stay in the current job in the next year was directly proportional to job satisfaction, corroborating with studies that evidenced that job satisfaction favors retention^(^ 25 ^)^ while dissatisfaction favors turnover at work^(^ 19 ^)^.

This study enabled the evaluation of job satisfaction of nursing professionals in critical care units. However, it presents some limitations related to the nature of cross-sectional studies, which makes it impossible to find existing causal relationships, as well as to generalize the data. In addition, it was performed in a single educational institution and the results found may differ from other ICUs.

The findings of this study suggest that implementing career plans and benefits, as well as readjustment of staff and improvement of working conditions may result in job satisfaction. In addition, this study may contribute to managers and administrators to develop strategies aimed at improving nursing professional conditions, with consequent repercussions on the quality of care and patient safety, thus improving institutional indicators.

## Conclusion

The nursing professionals, in general, reported ambivalence regarding job satisfaction. In the analysis by domains, they were satisfied with supervision, co-workers, and nature of work, and dissatisfied with contingent rewards, pay, benefits, operating procedures, and promotion.

The variables age, sex, professional category, work shift and type of ICU were associated with job satisfaction. Moreover, the shorter the time of experience in the unit and the institution and the greater the intention to stay in the job, the greater the job satisfaction.
